# HIV infection of non-dividing cells: a divisive problem

**DOI:** 10.1186/1742-4690-3-74

**Published:** 2006-10-26

**Authors:** Ariberto Fassati

**Affiliations:** 1 Wohl Virion Centre and MRC-UCL Centre for Medical Molecular Virology, Division of Infection and Immunity, University College London, 46 Cleveland Street, London W1T 4JF, UK

## Abstract

Understanding how lentiviruses can infect terminally differentiated, non-dividing cells has proven a very complex and controversial problem. It is, however, a problem worth investigating, for it is central to HIV-1 transmission and AIDS pathogenesis. Here I shall attempt to summarise what is our current understanding for HIV-1 infection of non-dividing cells. In some cases I shall also attempt to make sense of controversies in the field and advance one or two modest proposals.

## Background

DNA viruses and some RNA viruses must access the nucleus to replicate. This is also the case for lentiviruses and several excellent reviews have been recently published on the subject [1-5]. The interior of the nucleus is separated from the cytoplasm by a double-layer membrane contiguous with the ER called the nuclear envelope (NE) [6]. Embedded in the nuclear envelope are nuclear pores, which are channel-like macromolecules regulating access to the nucleus. In simple functional terms, nuclear pores can be considered like selective filters that allow diffusion of ions and molecules smaller than 9 nm across the NE and facilitated passage of larger molecules up to 39 nm in diameter if certain conditions are met [6]. To date, nuclear pores are the only known structures regulating ordered nucleocytoplasmic trafficking. Movement of proteins, mRNAs, tRNAs, rRNAs and nucleoprotein complexes in and out from the nucleus obeys to the selective biophysical nature of nuclear pores [7-11]. Viruses are no exception to this rule, thus understanding the structural and functional nature of nuclear pores is crucial to understand nucleocytoplasmic trafficking of viruses.

**Figure 1:**
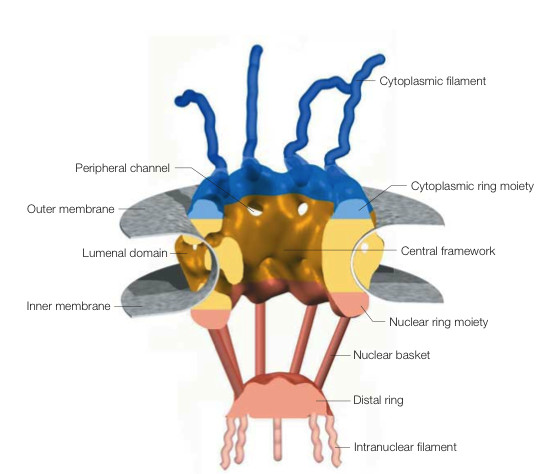
Three-dimensional structure of the nuclear pore complex. The main components of the pore include the central framework (yellow), the cytoplasmic ring moiety and attached filaments (blue), the nuclear ring moiety and the distal ring of the nuclear basket (orange). Nuclear membranes are depicted in grey. Reproduced with permission from Fahrenkrog B and Aebi U, Nature Reviews Molecular Cell Biology 4: 757–766 (2003) Macmillan Magazines Ltd.

Nuclear pores have a maximum diameter of 120 nm, a depth of 180 nm and an overall mass of approximately 125 MDa in vertebrates [6-12]. Recent 3-D images of *Xenopus Laevis *oocytes nuclear pores were obtained using energy-filtering transmission electron microscopy and tomographic 3-D reconstruction [13] (Figure 1). The nuclear pore appears to be constituted of two main ring moieties, a larger one facing the cytoplasm and a smaller one facing the nucleus. The two rings are joined together by a central framework, which is perforated by eight peripheral holes with a 10 nm diameter. The small holes are likely to be the site of ion and small molecules trafficking in and out from the nucleus. The central framework has eight external protuberances that presumably anchor the pore to the nuclear envelope. Flexible filaments approximately 50 nm long protrude from the cytoplasmic ring. Initially these filaments were thought to be important for the early steps of nuclear transport, by trapping and concentrating cargoes at the entry of the pore [14]. However, it has later been shown that depletion of the cytoplasmic filaments has only a modest effect on the overall efficiency and selectivity of nuclear import processes [15]. Eight filaments of approximately 75 nm in length depart from the nuclear ring and join the distal ring forming the so-called "fishtrap" or nuclear basket [6]. Nuclear pore complexes are composed of approximately 30 different proteins (called nucleoporins), some of which are integral and others are dynamically associated with the main structural scaffold [16]. Several large nucleoporins have phenyalanine-glycine rich domains (FG-rich). FG-rich domains are highly flexible and mobile, are mostly unfolded and thus able to interact with many binding partners simultaneously with fast association and dissociation rates [6]. FG-rich nucleoporins are thought to be essential to regulate kinetics and selectivity of nuclear import by constituting a selective gating or permeability barrier to molecules. Although the exact mechanisms are not understood and there are several proposed models [14,17-19], it is clear that nuclear import receptors, such as importins, mediate passage of their cargos through this mesh of FG-rich domains, possibly by conferring to the cargos themselves an overall mildly hydrophobic property and appropriate affinity for certain nucleoporins [18,19]. This might be accomplished by the mildly hydrophobic nature typical of all importins examined so far [19]. Importins would then act as "chaperones" for proteins translocating across nuclear pore complexes.

**Figure 2:**
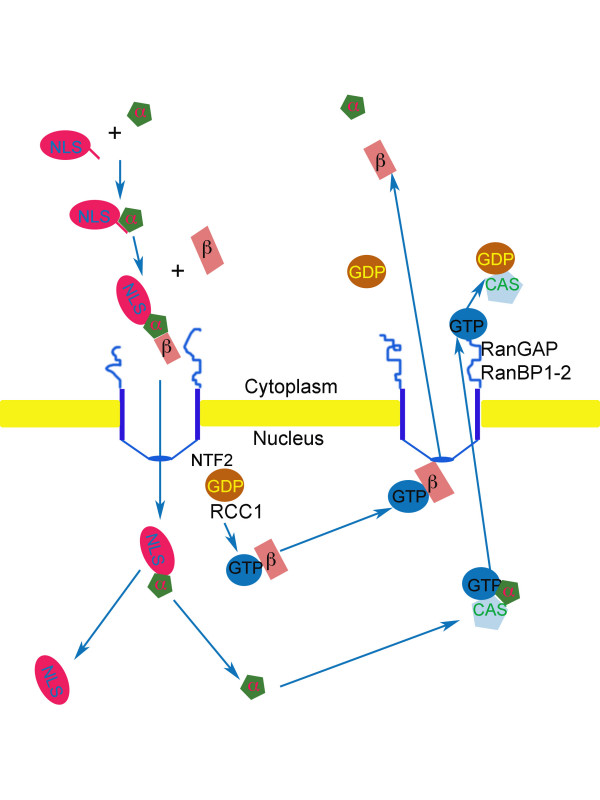
Schematic representation of the nuclear import cycle. The cargo (red) with a NLS binds impα and then impβ binds to impα forming a trimeric complex in the presence of RanGDP. The trimeric complex goes across nuclear pores and reaches the inner nuclear region. Here high levels of RanGTP induce a conformational change in impβ, which dissociates from the cargo and shuttles back to the cytoplasm. Impα binds to CAS in the nucleus and forms a complex with RanGTP. Such complex is exported from the nucleus and dissociates in the cytoplasm upon conversion of RanGTP into RanGDP. RanGDP is imported into the nucleus by NTF2. In the nucleus, RanGDP is rapidly converted into RanGTP by RCC1. At the cytoplasmic face of the nuclear pore, RanGTP in converted to RanGDP by RanGAP, RanBP1 and RanBP2. Thus a gradient of RanGTP across the nuclear envelope is maintained, which gives directionality to the import/export process.

Importins bind to their cargos through recognition of specific domains called nuclear localizing signals. The association or dissociation of importins from cargoes depends on the small GTPase Ran. In its GDP form, it promotes association whereas in its GTP form it promotes dissociation [7-9]. A gradient of RanGTP is maintained across nuclear pores by RanGAP1, RanBP1 and RanBP2 that induce hydrolysis of RanGTP into RanGDP at the cytoplasmic face of nuclear pores. Conversely, RCC1 converts RanGDP to RanGTP in the nucleus. NTF2 transports RanGDP into the nucleus for conversion by RCC1 and thus reconstitution of the nuclear RanGTP pool. So the transport cycle begins in the cytoplasm by binding of the appropriate importin to the cargo in the presence of RanGDP (Figure 2). The cargo can now be chaperoned across the nuclear pore complex. Once the cargo has reached the nuclear side, in the presence of RanGTP, the affinity of the importin for the cargo decreases dramatically and the cargo is released and trafficked to the appropriate nuclear compartment [7-9] (Figure 2). There are exceptions to this rule since some cargoes, like certain hnRNPs, are shuttled across nuclear pores in a Ran-independent way [20-22]. Although energy per se does not seem to be required for translocation across nuclear pores of simple cargoes *in vitro*, except for the generation of RanGTP, hydrolysable GTP and ATP may be required for nuclear import of large protein or nucleoprotein complexes *in vitro *and *in vivo *[23-25].

Interestingly, nuclear pore complexes are dynamic and respond to cellular stimuli. For example Ca^2+ ^induces conformational changes to nuclear pore complexes and the distal nuclear ring dilates in the presence of Ca^2+ ^or contracts in its absence. Thus the distal ring may function like an iris-like gate and regulate passage of cargoes [12]. The differentiation and proliferative state of the cell is also likely to play a role. Nuclear uptake rate for large particles is significantly higher in dividing cells compared to growth-arrested or serum-starved cells and terminal differentiation has been shown by electron micropscopy to increase both the efficiency of nuclear import and the size of imported particles through nuclear pores [26-28]. The fact that larger particles are imported into the nucleus of dividing cells with greater efficiency suggests that the permeability of nuclear pores may be subject to regulation, perhaps by partly changing the composition of the pores themselves. Moreover, nuclear import may also be modulated by phosphorylation and cell metabolism [29,30]. I propose that different regulation of the rate and overall permeability of nuclear import in dividing versus non-dividing cells and in differentiated versus undifferentiated cells is likely to be relevant to nuclear import of retroviruses, as it will be discusses in more detail later.

## The challenges facing viruses and the nuclear import system

Many viruses have to pass through nuclear pores to reach the nucleus, thus three general problems become immediately obvious. First problem, the capsid of many viruses, including adenoviruses, herpesviruses and retroviruses exceed the maximum diameter for passage through nuclear pores. Different viruses have evolved different systems to overcome this limit [31-34]. Some viruses, like adenoviruses, dock their partially disassembled capsids to the cytoplasmic face of nuclear pores, then the capsid completes disassembly by a process called uncoating, which leads to exposure of the viral nucleic acids to the nuclear import machinery. Other viruses, like herpex simplex virus, dock their capsids at the NE and eject the nucleic acids directly into or very close to nuclear pores. Yet other viruses uncoat in the cytoplasm and their genome engages with the nuclear import machinery at an earlier stage. It is likely that the overall structural stability of the intracellular viral capsid, the need to maintain a large genome tightly packed to facilitate cytoplasmic trafficking and the need to carry out enzymatic reactions (as in retroviruses) are all important factors in driving the evolution of different strategies to uncoat and overcome the size limit set by nuclear pores.

Second problem, regardless of how and where the uncoating step takes place, viral nucleic acids must be imported into the nucleus against a steep gradient, since nucleic acids are compacted to a very high density within the nucleus itself (nearly 0.1 g/cm^3 ^in lymphocytes) [35]. Some bacteriophages might have solved a similar problem of introducing their genome into the bacterial host cell by actively packing DNA into their capsid to a very high density with a pressure reaching 6 MPa. Such internal pressure is likely to provide sufficient force to inject the phage DNA genome into the bacterial cell at the time of infection [36]. However, there is no evidence so far that mammalian viruses use a similar mechanism to inject their genome into the nucleus.

Third problem, large nucleic acids molecules are charged and hydrophilic. As mentioned earlier, passage through the central channel of the nuclear pore complex depends on hydrophobic interactions with the highly mobile phenylalanine-glycine (FG)-rich domain of nucleoporins [6]. Thus, such large viral nucleoprotein complexes need to be somehow appropriately chaperoned to transit across nuclear pores.

## The interphase nucleus: not every retrovirus's club

Having broadly defined the general context and problems relative to nuclear import of viruses' genome in mammalian cells, let us now focus on retroviruses.

The ability of lentiviruses to infect terminally differentiated, non-dividing cells has been taken as proof that the genome of such viruses is imported into the nucleus. The inability of simple retroviruses to infect non-dividing cells on the other hand has been taken as proof that the genome of such viruses cannot be imported into the nucleus. The picture is not that clear-cut because different retroviruses infect non-cycling cells with different efficiency.

There is overwhelming evidence that lentiviruses infect non-dividing cells with high efficiency. Caprine arthritis-encephalitis virus (CAEV) has increased tropism for differentiated macrophages both in vitro and in vivo [37-39] and can infect dendritic cells [40]. Equine infectious anaemia virus (EIAV) is also found mainly in terminally differentiated macrophages of infected horses [41]. SIV and HIV infect differentiated macrophages, microglial cells and intestinal mucosa resting memory CD4+ T cells [42-51]. Clearly, the ability of lentiviruses to infect these types of non-dividing cells is crucial for virus spread and disease pathogenesis. Moreover, HIV-1 was found to be able to infect cells arrested in the cell cycle by treatment with aphidicolin or γ-irradiation and HIV-1 derived vectors infect hematopoietic stem cells and neurons [52-54]. In fact it appears that passage through mitosis is not a significant alternative pathway for HIV-1 infection even in dividing cells although nuclear envelope breakdown can modestly influence the kinetics of virus association with chromatin [55-57].

On the other hand there is strong evidence that gammaretroviruses can efficiently infect only cycling cells, with one notable exception [58-61]. The block to Moloney murine leukaemia (MoMLV) infection in non-dividing cells is up to 10,000 fold and it is independent of the specific phase of the cell cycle (i.e. G1/S or G2 or G0). The virus can synthesize linear full length DNA but cannot integrate or produce circular 2LTR DNA forms. Alpharetroviruses infect non-cycling cells more efficiently than MLV but less efficiently than HIV [62-67]. Foamy viruses also appear to have some ability to infect non-dividing or slowly dividing cells [68,69], for example they can infect human umbilical cord CD34+ cells and peripheral blood lymphocytes quite efficiently [70-72]. It is possible however that foamy viruses' ability to infect non-dividing cells depends on the long persistence of the pre-integration complex inside infected cells, until division eventually takes place [73].

So, why can lentiviruses infect non-dividing cells and gammaretroviruses cannot? The basis for this difference may reside in the uncoating process [2,74,75]. HIV-1 appears to shed its capsid shell quite early during infection, presumably in a manner that is timed with ongoing reverse transcription [76,77]. Evidence to support early HIV uncoating include the very low abundance of p24 CA protein found associated with the reverse transcription complex (RTC) and the PIC, the high density of the RTC/PIC in linear sucrose gradients, structural and morphological analyses of the RTC by electron microscopy, and the ability of RNA aptamers, siRNA and certain nucleoprotein complexes to interact with the incoming viral RNA genome [76,78-86] (although siRNA targeting of incoming viral RNA is not universally observed [87]). Clearly the viral capsid must remain viable for some time after infection since it is targeted by TRIM5α and related TRIM5s, which block reverse transcription, perhaps by anticipating the uncoating process itself [88,89]. Recent kinetic analyses suggest that the HIV-1 capsid remains viable for approximately 30–60 minutes following entry and after that it can no longer be targeted by TRIM5-CypA [90]. MLV, on the other hand, appears to maintain a capsid shell at least until nuclear entry, as shown by electron microscopy analyses of MLV-infected cells and biochemical studies of the RTC [91-93]. Indeed, substantial amounts of p30 CA are associated with MLV RTCs [92,93]. MLV RTCs synthesize full length viral DNA in the endogenous reverse transcription assay in the presence of small amounts of detergent, which presumably help permeabilize the capsid, and the interior of the MLV RTC can be accessed by small molecules but not by antibodies [92].

The viral capsid of both HIV-1 (broad end) and MLV has a diameter of 60 nm or greater and cannot go across nuclear pore complexes even by an active process. Thus early uncoating may be a necessary, albeit probably not sufficient, step for nuclear import. Two experimental lines of evidence seem to support this possibility. Chimeric viruses in which HIV-1 p24 CA protein has been swapped with MLV p30 CA are unable to infect cells arrested in the cell cycle, hence MLV CA appears to be a dominant-negative factor for nuclear import [74]. HIV-1 p24 CA mutant that fails to dissociate from RTCs also block virus replication at the level of both nuclear import and integration [75].

Although this is an appealing model to explain the phenotypic difference between HIV and MLV, there are some aspects that merit attention. It is likely that MLV is also targeted to the nucleus. For example, MLV mutants in the p12^gag ^protein synthesize normal levels of full length viral DNA but fail to form 2LTR circles, perhaps because they cannot associate properly with nuclear structures [94]. Moreover, MLV p30 CA can be SUMOlated and mutants in the p30 CA SUMO target region are blocked in the early phases of replication [95]. This is an intriguing observation because SUMO is involved in nuclear targeting of RanGAP1 to RanBP2, the constituent of the external filaments of the NPC [96]. It is therefore tempting to propose that MLV is actively targeted to components of the nuclear pores, which are known to bind to mitotic spindles during anaphase and early telophase [97]. This would then help tethering MLV PICs to chromatin following cell division. The *Fv1 *gene might directly or indirectly prevent such interaction between nuclear pore components and MLV PICs and hence prevent PIC nuclear retention, viral DNA circularization and integration.

A recent observation that apparently does not quite fit into the uncoating model for nuclear import suggests that MLV can infect differentiated, post-mitotic macrophages almost as efficiently as HIV-1 based vectors [61]. MLV infection of macrophages is limited to a short time window and it is unlikely to occur *in vivo*. Nevertheless one can still reconcile this finding with the uncoating model by postulating that NPC permeability in a selected population of macrophages may be greater than usual and allow active passage of macromolecules with a diameter of 60 nm. It will be interesting to test experimentally if this is indeed the case. As mentioned earlier, NPC permeability changes depending on the metabolic state of the cell, its differentiation and its ability to divide. Alternatively, MLV PICs may persist longer in macrophages until some degree of uncoating takes place allowing nuclear import.

## Nuclear import of HIV-1, facts and controversies

Uncoating of the viral capsid is likely to be a pre-requisite for nuclear import but specific signals and import factors are also likely to direct intracellular trafficking of the RTC/PIC. Several such nuclear localisation signals (NLS) have been identified in the HIV-1 PIC but no unequivocal picture has emerged yet. The first NLS within the HIV-1 PIC was reported in the N-terminal region of p17^gag ^MA and mutation of two lysines at position 26 and 27 was shown to block HIV-1 replication in terminally differentiated primary macrophages but not in proliferating cells [98]. These observations were quickly confirmed although it appeared that Vpr also influenced HIV-1 infection of non-dividing cells in addition to MA [80,99]. Moreover, phosphorylation of MA on a C-terminal tyrosine (Y^131^) was reported to induce MA incorporation into PICs (via binding to integrase) and to be essential for HIV-1 infection of non-dividing cells [100,101]. A more complex picture of MA phosphorylation was proposed later since mutation of MA Y^131 ^did not appear to have an effect on virus replication [102,103].

Subsequently, however, three studies could not confirm the presence of an NLS in MA [103-107]. The phenotype of HIV-1 mutants in the N-terminal MA NLS also remained controversial. Whilst such mutants were originally reported to be severely and selectively impaired in primary macrophages, later reports showed that they were moderately (2 to 15 fold) defective in both dividing and non-dividing cells using spreading assays and single-cycle assays [104,105]. Remarkably, HIV-1 mutants with large deletions of the MA N-terminal region or even lacking the entire MA (but retaining a short N-terminal myristoylation signal) have been shown to still be able to replicate in both dividing cells and macrophages, albeit at reduced levels [108].

More recently, MA has been reported to have a CRM1-dependent nuclear export signal (NES). Mutations in this NES at position 18 and 22 of MA were shown to cause nuclear localisation/retention of viral RNA and severely impair HIV-1 infectivity. Furthermore, the NES has been proposed to override a presumably masked NLS in the context of the p55^gag ^polyprotein [109]. It is somewhat difficult to reconcile these latter findings with earlier work showing that deletions of MA from residues 8 to 87 have a modest effect on HIV-1 replication [108], yet differences in the HIV-1 strains used might at least in part account for the different results. It is also interesting that the NLS and NES in MA would appear to be separated by 4 residues only [98,109], although a novel NLS in MA has been reported [110]. In summary, although there is no agreement on the existence of a NLS in HIV-1 MA [98,105-107,109,111], there appears to be some consensus that mutations in the N-terminal region of MA have a moderate effect on virus infectivity in macrophages as well as other dividing cell types. Since such an effect is also detected in single-cycle assays, it is likely to involve some early, post-entry event.

The viral protein R (Vpr) has been shown to play a role in HIV-1 nuclear import (for a recent review on this subject please refer to [112]). The general consensus is that Vpr is a karyophilic protein. Indeed Vpr localises to the nucleus when expressed on its own and also possesses at least two transferable NLSs that induce nuclear accumulation if fused to larger proteins like the maltose binding protein or β-galactosidase [113-118]. Vpr also localises to the nuclear envelope, possibly by direct engagement with nucleoporins [116-119]. The pathways used by Vpr for nuclear import are not completely clear. Vpr is a small protein of 96 aminoacids and contains no canonical (SV40 T antigen/importin β binding domain [IBB] NLS). The N-terminal region of Vpr contains a NLS, which can bind to importin α and nucleoporins [116,118] yet Vpr is not imported into the nucleus by the importin α/importin β heterodimer [116-118,120]. An additional trasferrable NLS has been detected in the C-terminal region (aa 73–96), which uses a pathway distinct from the N-terminal NLS. Both C- and N-terminal NLSs were shown to use a Ran-independent pathway and to require minimal energy [117]. It has been proposed that Vpr bypasses the normal requirements for nuclear import like Ran-dependent interaction with nuclear import receptors and instead can bind directly to nucleoporins, similar to importin β family members [117,118].

Importantly, Vpr is incorporated into virions at high levels via its interaction with the p6 domain of p55^gag ^[121]. The ability of Vpr to be incorporated into virions and enhance infectivity in macrophages might in addition depend on its nucleocytoplasmic shuttling properties and to a NES located in the C-terminal region, although this possibility is contentious and Vpr nuclear export may be more important in regulating cell-cycle arrest [122,123]. Vpr is retained in the RTC/PIC [76,80,83,124]. Thus, it has been proposed that Vpr stimulates docking of the RTC to the nuclear pores or alternatively disrupts nuclear envelope and pores to alter nuclear permeability [125,126]. Indeed many studies have shown that mutations in Vpr reduce HIV-1 ability to replicate in different cell types, including macrophages and PBMCs [80,116,118,127]. The replication defect is greater in cells infected a low MOI but is only a few fold. Consistent with this phenotype, HIV-1 based vectors that do not encode Vpr are still able to infect macrophages and other non-dividing cell types [128]. Moreover, Rhesus monkeys infected with SIVmac strains with a mutation in Vpx, which is the gene for nuclear import corresponding functionally to HIV-1 Vpr [129], showed lower virus burden, delayed decline in CD4+ counts but eventually progressed to AIDS [130]. Similarly, SIVsm strains mutant in Vpx also showed delayed replication kinetics in pigtailed macaques, possibly due to reduced virus amplification at early, post-transmission stages [131]. Taken together, these data suggest that the nuclear import properties of HIV-1 Vpr are probably not essential for virus replication but may increase HIV-1 infectivity and ability to propagate in specific cell types.

Integrase remains stably associated with the RTC and later the PIC and is the necessary protein for integration of viral DNA into host chromosomes. Because of its tight association with the PIC even after nuclear entry, integrase would be a good candidate to mediate HIV-1 nuclear import. HIV-1 integrase (IN) is karyophilic but there is some disagreement on the mechanisms regulating IN trafficking into the nucleus. Most investigators have reported that IN is imported into the nucleus by an active, saturable and energy -dependent mechanism. Putative NLS have been mapped to several regions of the C-terminus of IN [111,132-134] and to the central catalytic domain [135,136], although some of these putative NLSs have been questioned [133,137,138]. Moreover, IN can bind to importin α [25,111,138], to importin β directly [25,138] as well as to importin 7 and transportin [25]. At least importin α, importin β and importin 7 have been shown to stimulate nuclear accumulation of IN in a Ran-dependent way [25] and in one recent study blocking antibodies against importin α and/or importin β were shown to reduce IN nuclear import [138]. Antibodies against importin 7 did not block IN nuclear import in one study [138], however it is not clear which anti-imp7 antibodies have been used in that study and to our knowledge antibodies with good affinity for native importin 7 are not available. Therefore those negative results should be interpreted with caution. Interestingly, it has been recently reported that HIV-1 Rev binds to and is imported by a number of importins, including importin β, importin 7 and transportin [139]. The adenoviral pVII protein, which is tightly bound to the viral DNA and is thought to mediate nuclear import of the adenoviral genome, has also been shown to bind to and be imported by the impα/β heterodimer, impβ alone, imp7 alone, imp7/β heterodimer and transportin [140]. Thus adenoviral pVII protein and HIV-1 IN appear to behave in a remarkably similar way [25,140]. Adenoviral pVII and HIV-1 Rev and IN are all small, basic nucleic acids-binding proteins and may share similar nuclear import pathways. Moreover, relying on multiple importins may give a selective advantage to viruses by maximising nuclear import efficiency in different conditions and cell types. It is plausible that some ribosomal proteins have adopted a similar strategy to ensure their efficient nuclear import [141].

Lentiviral INs specifically bind to lens epithelium-derived growth factor (LEDGF/p75), a hepatoma-derived growth factor that interacts with DNA and this association has been reported to be important for IN nuclear localisation [142,143]. IN and LEDGF/p75 have been shown to co-localise in nuclei following transfection of plasmids encoding for the two proteins. Mutant IN unable to interact with LEDGF/p75 was shown to have lost its ability to accumulate into nuclei and siRNA-mediated knock down of LEDGF/p75 induces re-localisation of exogenously expressed IN to the cytoplasm [143-146]. These data point to the possibility that LEDGF/p75 mediates nuclear import of IN. However, pre-incubation of LEDGF/p75 with IN did not stimulate IN nuclear import in the nuclear import assay [145]. Severe knock down of LEDGF/p75 inhibits HIV-1 infectivity at the level of integration but not appreciably at the level of RTC nuclear import and the nuclear rather than the cytoplasmic pool of LEGDF/p75 appears to be the important for HIV-1 replication ([147]; E. Poeschla personal communication). Since IN is readily ubiquitinated in stably expressing cells lacking LEDGF/p75 [144], such modification might cause loss of IN nuclear import.

Is it possible to reconcile these findings with the observation that, at least in vitro, IN can be imported into the nucleus by an active mechanism in the absence of LEDGF/p75? It is perhaps interesting that the integrase-binding domain in LEDGF/p75 is structurally closely related to the HEAT repeat found, amongst other proteins, in importin β and other importins [148]. It is therefore tempting to speculate that the very same region in IN mediating LEDGF/p75 binding also mediates interaction with importins via the HEAT motif. If this is the case, then alternative binding of LEDGF/p75 and importins might take place. Importins binding to IN might prevail in the cytoplasm due to their relative abundance in this compartment whilst LEDGF/p75 binding to IN might prevail in the nucleus, following dissociation of importins from IN in the presence of RanGTP. Then LEDGF/p75 might tether IN to chromosomes and stimulate HIV-1 DNA integration. A prediction of this model is that LEDGF/p75 competes with importins for binding to IN in the absence of RanGTP though one would expect LEDGF/p75 to bind to IN with greater affinity than importins.

Alternatively, lentiviral INs per se might not have a transferable NLS as shown by two reports [149,150] and nuclear accumulation could simply be the result of diffusion across nuclear pores, DNA binding and nuclear retention. LEDGF/p75 tethers IN to chromosomes, hence it might appear to induce nuclear import.

Finally, a few words of caution should be spent on the role of IN in HIV-1 nuclear import. There is limited information on the precise conformation of IN within the RTC/PIC and several domains, which are exposed in the recombinant protein, might not be available once the protein is part of the RTC/PIC and bound to nucleic acids [132,151]. Moreover, it is now clear that IN serves multiple roles in addition to viral DNA integration and that mutating putative IN NLSs may result in unexpected phenotypes unrelated to nuclear import [132,152-156]. Thus, it has proven a rather difficult task to translate results obtained *in vitro *by mutagenesis of IN into a clear phenotype of reduced HIV-1 nuclear import. Remarkably, a recent study has analysed the phenotype of HIV-1 chimeric viruses bearing MLV IN in place of HIV-1 IN and shown that such mutants are attenuated but still able to infect non-dividing cells. Only a small increase was observed in the ratio of cytoplasmic to nuclear viral DNA in cells infected with the mutant virus compared with wild-type HIV-1 [157]. Thus, it is possible that IN does not play an important role in HIV-1 nuclear import, although it should be noted that MLV IN has also been shown to rapidly accumulate into the nuclei of infected cells [91].

The central polypurine tract (cPPT) was also shown to influence HIV-1 nuclear import. The cPPT is a second origin of DNA plus strand synthesis located in *pol*, it is present in all lentiviruses and results in a short (approximately 100 nt) stretch of triple stranded DNA upon completion of reverse transcription [158,159]. Absence or mutation of the cPPT was shown to abolish HIV-1 replication in a spreading assay and to reduce infectivity by 5–7 fold in single cycle infection assays using HIV-based vectors. Reduced 2LTRs circular viral DNA formation and hence nuclear import was reported to be the main defect of the mutant viruses [160,161]. This observation has been confirmed by many studies using HIV-1 based vectors in several cell lines and primary human cells, including PBMCs, T-lymphocytes, macrophages, CD34+ cells and in rat neurons [162-165]. Indeed it is now standard practice to include the cPPT element in the design of "second and third generation" lentiviral vectors. A few features of the phenotype of cPPT+ vectors are worth noting. First, the cPPT+ vectors have increased infectivity in both dividing and non-dividing cells. Second, cPPT+ vectors appear to have an increased rate rather than an absolute increase of HIV-1 DNA nuclear transport. Third, there seem to be a slight increase in integration efficiency with cPPT+ vectors and fourth, cPPT+ vectors are able to overcome a saturation effect seen with cPPT- vectors, hence they work better at high MOI [162-165]. This would lend support to the hypothesis that the cPPT can "boost" HIV-1 vector infection bypassing (partially) the requirement for some limiting cellular factor important for nuclear import.

The picture becomes more complicated when wild type viruses with a normal or mutated cPPT are tested in spreading assays. In this case a modest attenuation of cPPT- viruses is seen only in some cell types and even in single cycle assays the difference between cPPT- and cPPT+ viruses' infectivity has been reported to be approximately two fold [153,154]. A similar degree of inhibition has been observed with the yeast Ty1 retrotransposon lacking the cPPT element [166]. The reasons for this discrepancy are not completely clear. One simple explanation would be that virus growth in spreading assays is generally measured by p24^gag ^ELISA or by enzymatic RT assays and both assays have higher variability than GFP detection by FACS in single cycle assays. Thus, if the variability of the detection assay is greater than the experimental difference to be observed, the results cannot be accurately measured. Alternatively, a difference in the rate rather than absolute amount of HIV-1 DNA nuclear import may not be detectable in spreading assays due to the highly asynchronous infection process, as opposed to a more synchronous infection with viral vectors. Elements in the HIV-1 genome, not included in viral vectors, might also partially compensate for the lack of the cPPT. Recently, a more severe replication defect, consistent with the one found with HIV-1 based vectors, has been reported for different HIV-1 strains lacking the cPPT element in single cycle assays and spreading assays [167]. Even if the cPPT may not be absolutely essential for HIV-1 replication, at least in tissue culture, this viral element remains a very interesting biological phenomenon and also has important practical applications in gene therapy.

## Cellular factors

**Figure 3:**
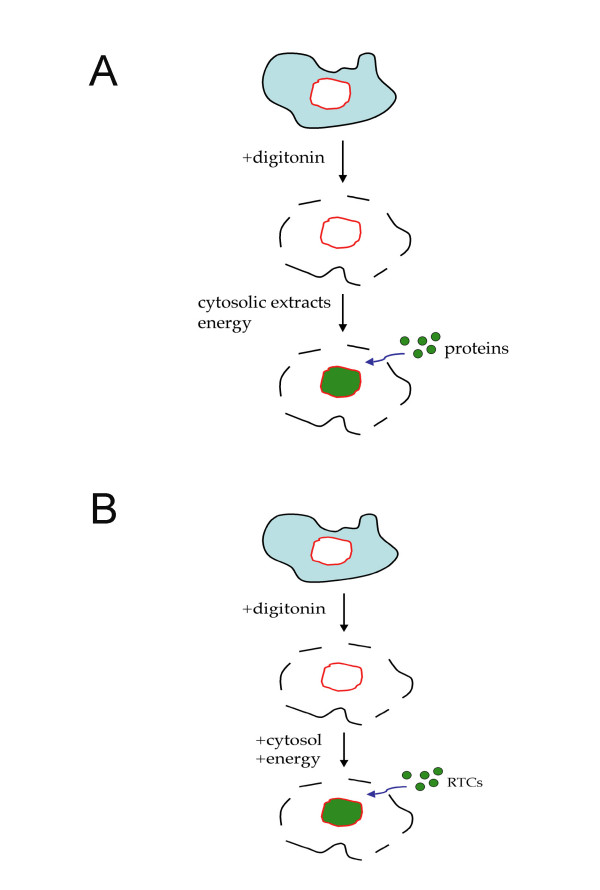
Schematic representation of the nuclear import assay. (A) The classic assay. Cells are permeabilized with digitonin, which leaves the nuclear envelope intact. The cytosol is washed out and nuclear import is reconstituted by addition of the protein of interest (labelled fluorescently), cytosolic extracts or nuclear import receptors, the Ran system (RanGDP, NTF2, RanGAP, RanBP1) and an energy regenerating system. Accumulation of the protein of interest is examined by confocal microscopy. (B) Adapted assay. The assay is carried out as described above but purified and labelled HIV-1 RTCs are used instead of the protein of interest.

Mutations of all known viral karyophilic elements does not block HIV-1 replication [157], suggesting that viral components are unlikely to be the only factors regulating HIV-1 nuclear transport and that cellular factors need also to be investigated. One major problem in this case was the lack of a convenient *in vitro *assay to screen for potential cellular factors with HIV-1 nuclear import ability. The field of nuclear import has advanced at an impressive pace since the introduction in the early '90s of the so-called "nuclear import assay" [168] (Figure 3). In this assay, the cell plasma membrane is permeabilized with digitonin, which solubilises cholesterol and hence leaves the nuclear envelope intact. Soluble intracellular components are washed out in buffer and nuclear import is artificially reconstituted by the addition of the fluorescent-tagged substrate of interest, cytoplasmic extracts or specific cellular factors, the Ran system and an energy regenerating system. The cells are incubated at 25 to 37°C for a short time, fixed and then analysed by confocal microscopy. Nuclear accumulation of the tagged substrate is detected when the right cellular components are added. Thus, this assay has allowed screening for many factors and has led to the identification of several importins or karyopherins [168-171].

We have adapted this assay to investigate cellular components involved in HIV-1 nuclear trafficking (Figure 3). Purified HIV-1 RTCs are fluorescently labelled and added to the permeabilised cells, which can be HeLa or primary macrophages. This approach does not depend on mutations of viral proteins and putative nuclear import components can be tested individually, thus multiple and potentially redundant import pathways can be identified and dissected.

Using this approach we have recently found that importin 7 (imp7) stimulates nuclear import of HIV-1 RTCs and that siRNA-mediated depletion of imp7 inhibits HIV-1 infection, though only by a few fold [25]. Another study, however, using the siRNA approach, failed to see a phenotype in imp7 knock down cells and primary macrophages [172]. It should be noted that conflicting results were also reported for RNAi-mediated knock down of LEDGF/p75, which have cast doubts on the biological relevance of this proteins for HIV-1 replication [143,147,173]. A more radical knock-down of LEDGF/p75 has recently been reported to significantly affect viral replication, highlighting the fact that even small amounts of cellular factors are often sufficient to support normal HIV-1 replication (E. Poeschla, personal communication). The unambiguous role of LEDGF/p75 in HIV-1 replication is supported by recent LEDGF/p75 knock out studies (A. Engelman, personal communication). We have since observed that the efficiency HIV-1 but not SIVmac infection is reduced in stable imp7 shRNA knock down cell lines (Fassati et al. unpublished). However, the development of effective imp7 dominant negative mutants or cells with a conditional imp7 knock out will probably be needed to investigate the full impact of this protein on the replication of HIV-1 and other retroviruses.

More recently we seek to identify additional cellular factors involved in nuclear import of HIV-1 RTCs. Using chromatographic procedures and the nuclear import assay we have isolated a near-homogeneous fraction from cytoplasmic extracts. This fraction contained tRNAs, most of them with defective 3' CCA ends. When synthesized *in vitro*, such tRNAs promoted HIV-1 RTC nuclear import. Moreover, tRNAs with RTC nuclear import activity were incorporated into and recovered from virus particles. We found that the anticodon loop mediated binding to the viral complex whilst the T-arm may interact with cellular components involved in nuclear import. These tRNAs species were transported into the nucleus on their own in a energy- and temperature-dependent way. We also observed that HIV-1 mutant containing MLV *gag *[74] did not incorporate tRNA species capable of promoting HIV-1 RTC nuclear import and were impaired in infecting cell cycle-arrested cells [174]. Thus, by investigating HIV-1 nuclear import, we have found evidence for retrograde tRNA transport in mammalian cells, an unexpected process that has also been recently described in yeast [175,176]. Future work will hopefully elucidate which cellular factors participate in these events and whether the biological function of tRNA retrograde transport in mammalian cells is to modulate protein synthesis or is a tRNA quality control mechanism or both.

## Conclusion

Elucidating the mechanisms of HIV-1 nuclear import is clearly a challenging area of research, both from a technical and a conceptual point of view. It is also a promising area of research, likely to reveal new and fundamental cellular pathways. To gain more insight and perhaps a little inspiration, it may be wise to look at similar processes occurring in cells, like mRNA export. Export of mRNA nucleoproteins (mRNPs) involves several factors. One of the best characterized is TAP or nuclear export factor 1 (NXF1) [177]. At least six other members of the NXF family have been described, some having little export activity, others having an mRNA-specific or cell type-specific export activity [177]. Interestingly, despite their sequence and structural similarity, different NXFs can use different export pathways and different modes to bind to NPCs [178]. NXFs need adaptors to engage with NPCs. One such adaptor is p15, which allows TAP bindng to nucleoporins. NXFs need adaptors also to bind to mRNAs. The so-called REF protein is an adaptor for TAP but additional adaptors include several exon-exon junction complex proteins. Moreover, the structure, shape and maturation stage of mRNAs influence their export rate and ATP-dependent motor proteins are required, probably at more than one stage [177]. The level of complexity and sometimes redundancy typical of mRNA export is well suited to illustrate the point: HIV-1 nuclear import is very likely to involve a similar or higher degree of complexity.

In conclusion, I would now like to propose a model on HIV-1 nuclear import. Many parts of this model are still hypothetical; nonetheless I shall be bold enough to put it to the attention and critical mind of the reader. After entry, HIV-1 starts reverse transcription and shortly thereafter sheds, partially or completely, its capsid. Such shedding is sufficient to expose the nucleoprotein complex or RTC, composed of the viral genome (presumably still in part RNA) and some viral and cellular proteins. The RTC then engages with the nuclear import machinery at several levels. There might be adaptors, both viral (for example Vpr) and cellular (for example tRNAs), that promote RTC docking and binding to the nuclear pores. Once at the nuclear pore, additional signals/factors may facilitate the charged and hydrophilic nucleic acids to cross the pore's central channel (for example imp7), and other elements may recruit putative motors at the pore to overcome the steep DNA concentration gradient in the nucleus (for example the cPPT element?). RTCs are likely to undergo substantial conformational changes at different stages (RTCs convert from RNA into double stranded DNA) and viral and cellular factors are also likely to associate and dissociate dynamically from the RTC. The multiplicity of signals ensures that the rate of nuclear transport is fast and individual signals may predominate in specific cell types. Like a good orchestra, the loss of one element will reduce the quality of the performance but, depending on the element lost, it may be noticeable only to the educated ear. It will be important to identify possible bottlenecks in this process to develop effective anti-viral strategies. MLV, on the other hand, may not be able to shed enough capsid to make its RTC fully visible to the nuclear import machinery and/or may lack sufficiently strong NLS [179]. MLV may be docked close to or at nuclear pores but then it may have to wait patiently until it is tethered to chromatin only after dissolution of the nuclear envelope.

There is of course a fundamental question worth considering: why mammalian cells have an evolutionary conserved mechanism to import DNA into their nuclei? Perhaps the study of nuclear import of viral genomes will shed some light on this problem too.

## Abbreviations

HIV-1, human immunodeficiency virus type 1; SIV, simian immunodeficiency virus; MLV murine leukaemia virus; RTC, reverse transcription complex; IN, integrase; NPC, nuclear pore complex; cPPT, central polypurine tract; LEDGF, lens ephitelial-derived growth factor; MA, matrix protein; CA, capsid protein; mRNP, messenger RNA ribonucleoprotein.

## Competing interests

The author(s) declare that they have no competing interests.
